# Disease surveillance using online news: an extended study of dengue fever in India

**DOI:** 10.1186/s41182-019-0189-y

**Published:** 2019-12-11

**Authors:** Yiding Zhang, Motomu Ibaraki, Franklin W. Schwartz

**Affiliations:** 1 Environmental Science Graduate Program, The Ohio State University, 275 Mendenhall Laboratory, 125 South Oval Mall, Columbus, OH 43210 USA; 2 School of Earth Sciences, The Ohio State University, Columbus, USA

**Keywords:** Disease surveillance, Newspaper, Text mining, Dengue fever, India

## Abstract

**Background:**

The study demonstrates the potential in using newspaper information as a proxy for monitoring dengue fever outbreaks in India. Online newspapers are being considered as sources of information on disease surveillance, early outbreak detection, and epidemiology research. Our objective is to understand the complex dengue epidemiology and discover inter-relationships between dengue fever and local social-environmental factors by mining information from local Indian news articles.

**Results:**

We search and extract articles from the newspaper database, LexisNexis. News articles related to dengue fever in India are analyzed together with local environmental, climate, and population data in both temporally and spatially to study disease epidemiology. We also examine the influence of newsworthiness for constructing a disease surveillance system. In terms of temporal aspects, dengue outbreaks follow consistent patterns every year. However, for many areas, this application is frustrated by the relatively small numbers of news articles.

**Conclusions:**

The study has advanced capabilities in producing approaches that provide for richer interpretations of textual information provided in newspaper articles. Such approaches appear particularly well suited for developing countries with relatively poor medical infrastructures and records.

## Background

News articles have significant potential in providing information relevant to disease surveillance, early outbreak detection, and epidemiological research [1]. Although the content of articles in newspapers is usually influenced by many factors, such as the background of the journalists, and particular social issues, such information is still particularly helpful, especially in developing countries and regions with a relatively poor medical infrastructure and records. In a previous paper, we showed newspaper articles to be useful in providing proxy data capable of monitoring outbreaks of certain infectious diseases, including dengue fever in India and Zika in Brazil [2]. More specifically, this information helps in understanding temporal transmission trends in diseases, as well as the seriousness of outbreaks.

However, beyond the fundamental information on occurrence derived from straightforward word searches, e.g., numbers of news articles as proxies for numbers of disease cases, there is a diverse array of information represented by the written content of the articles. The information contained there can be identified and extracted using data mining techniques. Several approaches have been initiated and developed in the previous studies [3–9]. Our study examines the possibility of using such information to describe the epidemiology of infectious diseases. For example, specific information reported in articles on geographic locations, environmental factors, climate details, populations, social communities, etc. can be used to characterize diseases more completely. Clearly, if information from news articles can be extracted and developed productively, this approach represents a valuable contribution to data needed in studying poor developing countries and regions.

Dengue fever is used as an illustrative example of a disease for this study. This mosquito-borne viral disease commonly occurs in tropical countries and produces a significant public health burden, especially during epidemics. According to Murray et al., dengue fever is known to be endemic in 125 countries with case numbers between 50 and 200 million [10]. However, Brady et al. estimated 390 million “apparent and inapparent” infections [11]. Dengue affects large patient populations, a small percentage of which suffer from more serious forms including dengue hemorrhagic fever and dengue shock syndrome [12].

Dengue fever is associated with four dengue viruses (DENV-1, DENV-2, DENV-3, and DENV-4) [13]. It is transmitted primarily by a bite from an infected *Aedes* mosquitoes, particularly *A. aegypti*. Although humans are the primary host of the virus, dengue also circulates among nonhuman primates [14]. Case numbers have increased significantly in tropical and subtropical countries in recent years, in step with urbanization of the tropics that comes along with slum housing and with associated unhygienic conditions that foster the development of mosquitoes. Guzman and Istúriz estimated that about 2.5 billion individuals or approximate 40% of the world’s population, inhabit areas where there is a risk of transmission of dengue fever [15].

Our previous paper [2] discussed in detail the pros and cons of selecting well-known international newspapers versus popular local newspapers as information sources. We concluded that local newspapers provide the best sources of information with respect to dengue outbreaks in specific countries or regions. We also described the strong correlation between numbers of dengue fever cases and number of associated news articles from local major newspapers (The Times of India (TOI) and Hindustan Times (HT)). In other words, the numbers of news articles concerned with dengue fever serve as a proxy to predict temporal variability in dengue case numbers for India [2].

There is no disputing the impact that dengue fever has on India. This disease has been endemic and a severe health problem for more than 50 years [16]. Although government sources in India reported an annual average of 20,474 dengue cases between 2006 and 2012 [17], it is likely that this number is a substantial underestimate. Milder cases of dengue fever are unreported and patients with more severe dengue hemorrhagic fever and dengue shock syndrome may not have had access to hospitals.

The population of India is large, exceeding 1.3 billion people. The rapid growth in population and urbanization all favor the expansion of dengue geographically [10, 18]. Poverty, slum housing, and unhygienic conditions all contribute to an inability to control mosquitoes. A second reason why dengue fever is endemic relates to the climate. As a tropical country, India experiences a monsoon season every year, which yields most of the precipitation recorded in any given year [19, 20] (Additional file 1: Figure S3). The monsoons lead to preferable conditions for mosquitoes and their population increases significantly, hence, amplify dengue outbreaks [21, 22]. Additionally, temperatures over much of India are high throughout the year, which favors mosquitos [23].

The health impacts of dengue and the large numbers of affected people make the disease newsworthy. News reports usually focus on the intensity of outbreaks, numbers of dengue cases, their spatial and temporal distributions, mosquito activities, government activities, and dengue diagnoses and studies [2]. In comparison to well-known international news outlets (e.g., The New York Times, Cable News Network), news articles published locally contain a larger and more varied quantity of information associated with dengue fever. India has benefited from a robust and well-developed national network of news outlets (e.g., TOI and HT). The timely, well-organized, and systematic coverage from these sources is available online. Daily reporting provides a large and diverse collection of news articles, which covers a broad selection of topics related to dengue. These characteristics of local newspapers make them useful in applications concerned with exploring the complex epidemiology of dengue fever.

With this follow-on study, our goal is to expand the potential usefulness of news information not only to track disease cases through number of news articles, but also to take a step towards understanding disease epidemiology through detailed textual information. The specific objective here is to discover inter-relationships between dengue fever and local social-environmental factors (e.g., climate) by mining information from local Indian news articles and further to explore the practicality of applying news articles to study the details on the regional distribution of disease cases.

## Materials and methods

### Online newspaper database

Important national or local newspapers provide a better source of information on diseases in particular countries than key international outlets [2]. Accordingly, we created a process to identify suitable sources. LexisNexis emerged as the most suitable online news database because of its broad coverage of articles from thousands of outlets around the world, including India. Additionally, LexisNexis provides fully texted articles and well-formatted text data, which facilitate various applications in data mining [24]..

### Information extraction from news articles

Information downloaded from LexisNexis yields two collections of information, (1) data from 2010 to 2017 on the number of news articles from each of TOI and HT, two of the most important and popular national newspapers, and (2) textual information for each article from 2013 to 2016, extracted from the original articles. Key article features (e.g., title, the news article itself, authors, and published date) are selected and formatted for future use in text mining. The articles themselves were saved as plain text files.

The search technique that is used to identify news articles related to dengue fever is known as a Boolean logic search [25]. To be selected, a news article must contain the specified disease name, in this case “dengue” in the title and the specified country name (i.e., “India”) in any part of the article.

Another way we used the information in news articles was to identify particular geographic locations that were mentioned therein. A Python-based tool-kit was developed to extract information from the articles. We use this information to assess the possibilities for using spatial variability in article numbers to track spatial patterns in disease variability. To facilitate this analysis, we created text files that contained the names of states, union territories, regions, and cities for India. These files are essentially libraries to identify, extract, and count location names appearing in each newspaper article.

### Other needed data for India

In order to develop information on dengue fever outbreaks, data on population densities for the states and territories were obtained from Census of India 2011 [26]**.** India has an area of 3.29 million square kilometers and is subdivided into 29 states and 7 territories (refer to Additional file 1: Basic Information on India). There are eight states with population densities greater than 500 people/km^2^, including Kerala, Tamil Nadu, Punjab, Haryana, Delhi, Uttar Pradesh, Bihar, and West Bengal. Three states, Delhi, Bihar, and West Bengal, have population densities exceeding 1000 people/km^2^. Most states with the highest population densities are located around Delhi. Please refer to Additional file 1: Figure S2 for details.

Climatic factors, such as rainfall, temperature and relative humidity, are commonly associated with dengue fever outbreaks in India. For example, Chakravarti and Kumaria pointed out that the outbreaks coincided mainly with the post monsoon period when rainfall has declined [27]. The likely reason is that mosquito populations were able to increase substantially with increasing availability of standing water [21, 28]. In order to examine the effect of rainfall on dengue fever outbreaks, we gathered and compiled monthly and yearly rainfall data for India from 2013 to 2016 from Rainfall Statistics of India published by the India Meteorological Department, Ministry of Earth Sciences [29–32].

Other non-climatic factors, such as land use and vegetation, can affect mosquito population dynamics [33], but here, we only examine rainfall as the main factors because it is the major factor that affects yearly changes in mosquito populations, especially in a tropical country like India. Further, we assume that non-climatic factors are constant throughout our study period [23, 34].

## Results

### Association of dengue fever with monsoons

In our previous study, we used 7 years of data to establish a relationship between numbers of news articles from India concerned with dengue fever and yearly numbers of dengue cases [2]. The results demonstrated a statistically strong, non-linear, correlation between the actual numbers of dengue cases and newspaper reports concerned with dengue. Indications are that the frequency of news reports from national newspapers provides a useful proxy for examining multi-year trends in dengue transmission [2].

**Fig. 1:**
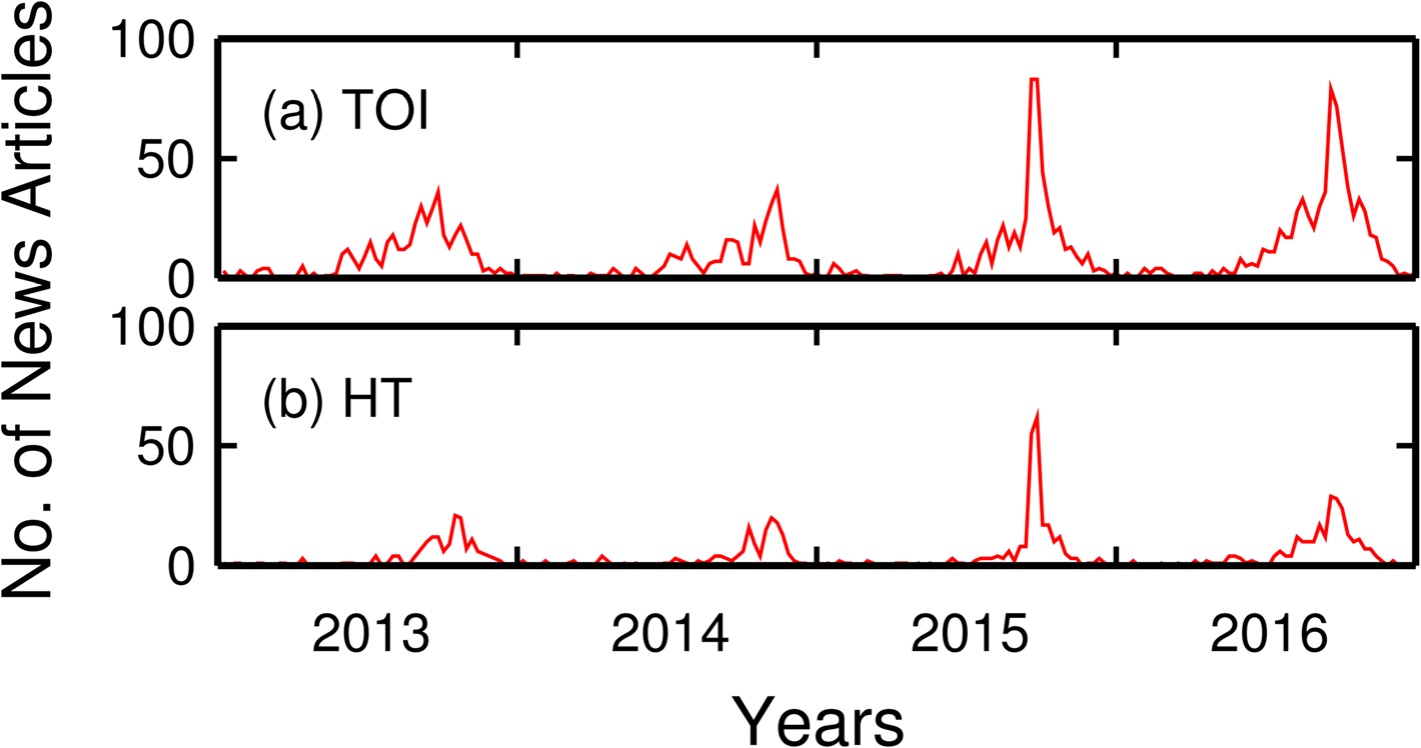
The time variation in the number of weekly news reports from Indian local news outlets from 2013 to 2016. **a** TOI and **b** HT

Figure 1 displays 4 years (2013–2016) of weekly numbers of news reports (TOI and HT) related to dengue plotted with respect to time. These results suggest that the number of dengue cases was relatively low in 2013 and 2014. However, 2015 and 2016 saw a surge in case numbers. Independent data from two sources [35, 36] showed that on annual basis the dengue cases numbers for these latter 2 years, 99,913 and 90,277, respectively, were the highest on record.

Another important trend evident in Fig. 1 is that the numbers of articles varied seasonally during a calendar year. Article numbers increase sharply between June and July, reaching a peak in late September to early October, before waning towards the end of each year. This trend matches the known behavior in the numbers of dengue cases. The numbers of dengue patients become noticeable in July, reaching a peak in September and October, before declining through November [2, 37].

**Fig. 2:**
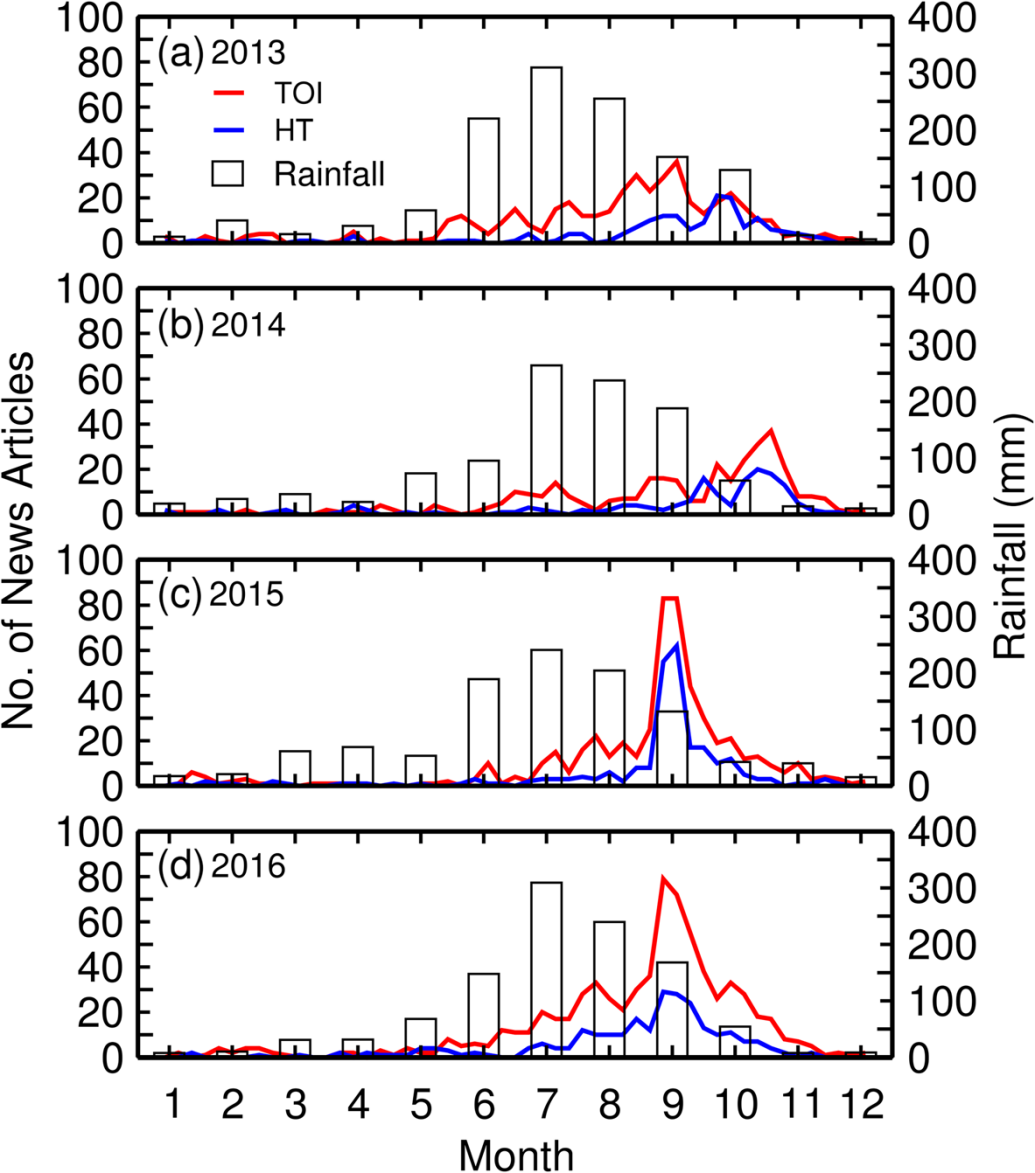
The number of news articles on dengue fever published weekly in TOI and HT from 2013 to 2016 compared with the average monthly rainfall for India

Figure 2 compares the number of news reports with monthly precipitation data to elucidate the timing of dengue outbreaks in relation to the summer monsoon season. In 2013 and 2014, the numbers of news articles were relatively small, which agreed with the small numbers of dengue cases. The years 2015 and 2016 were associated with the worst outbreaks of dengue fever since 1998. As expected, the numbers of news articles increased, as can be seen in Fig. 2c and d. In total for 2015 and 2016, TOI and HT published 1243 and 503 articles, respectively. Typically, the monsoon season begins in June with precipitation peaking in July. The number of dengue-related articles plotted versus time follows a similar pattern with a sharp peak in September, which lagged the peak rainfall by 2 months. The peak in numbers of articles from HT in 2013 as well as in 2014 behaved somewhat differently, exhibiting a 3-month time lag with peaks in October.

### Analysis of spatial patterns on the distribution of dengue fever in India

**Fig. 3:**
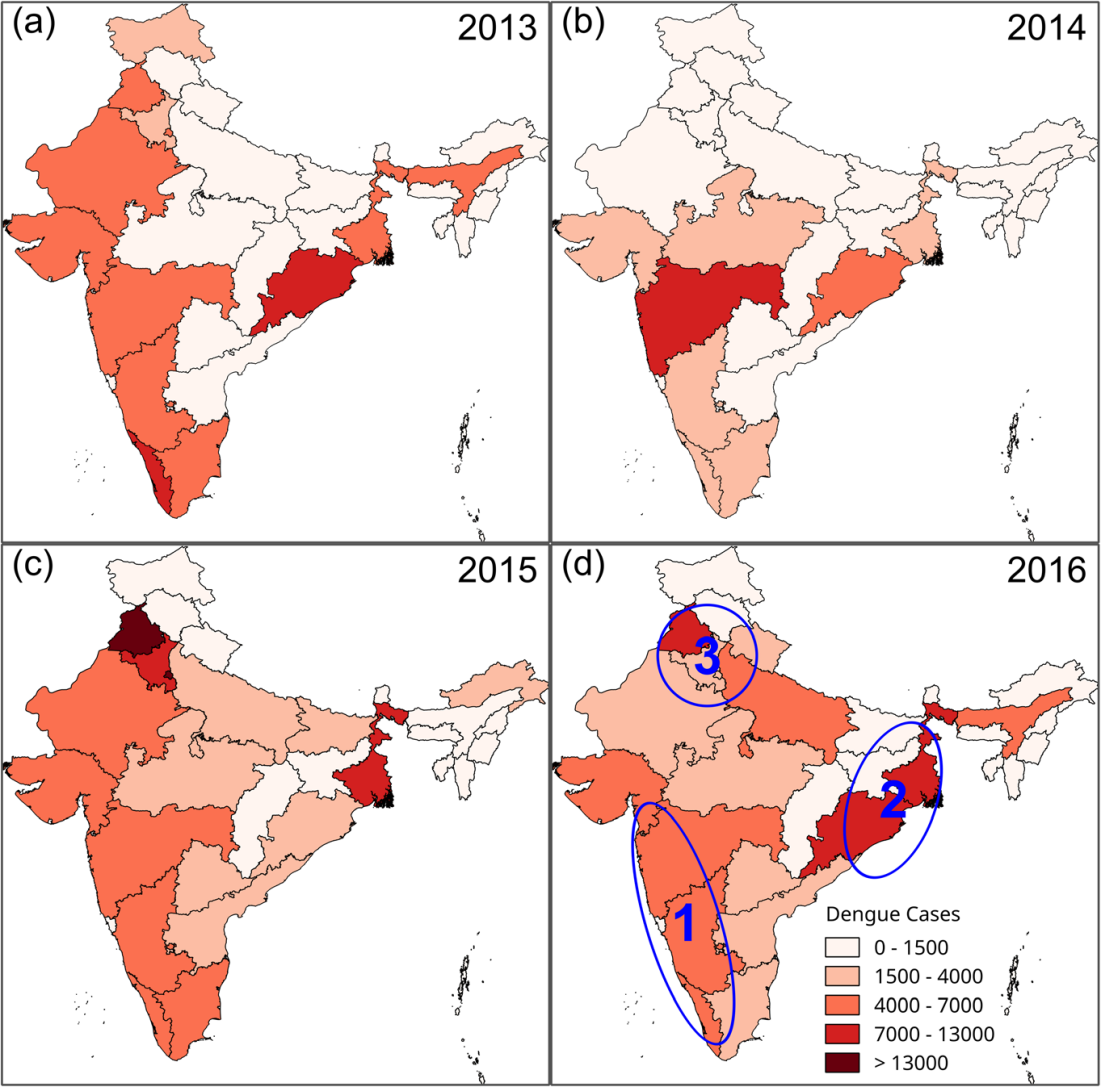
The shaded maps display state-wise variations in annual dengue cases from 2013 to 2016. Three areas consistently impacted by dengue fever are outlined in blue

This section examines the spatial variabilities in case numbers of dengue fever. A map of Indian states and union territories is shown in Additional file 1: Figure S1 (for additional information, refer to Additional file 1). Monthly numbers of dengue cases for each Indian state from 2013 to 2016 were obtained from the Indian Ministry of Health and Family Welfare [38] and plotted as annual totals for the states on the maps in Fig. 3.

The four maps (Fig. 3a–d) suggest that there are three areas that were commonly impacted by dengue outbreaks (areas circled in blue in Fig. 3d), (1) a long western coastal area (Area 1 in Fig. 3d), which includes the states of Gujarat, Maharashtra, Karnataka, Kerala, and Tamil Nadu; (2) a northeastern coastal area (Area 2 in Fig. 3d), which includes the states of West Bengal and Odisha; and (3) states around Delhi (Area 3 in Fig. 3d), which include Delhi, Uttar Pradesh, Haryana, and Panjab. We will explore these spatial patterns in greater detail in the “Spatial patterns inferred from numbers of newspaper articles” section.

### Spatial patterns inferred from numbers of newspaper articles

**Fig. 4:**
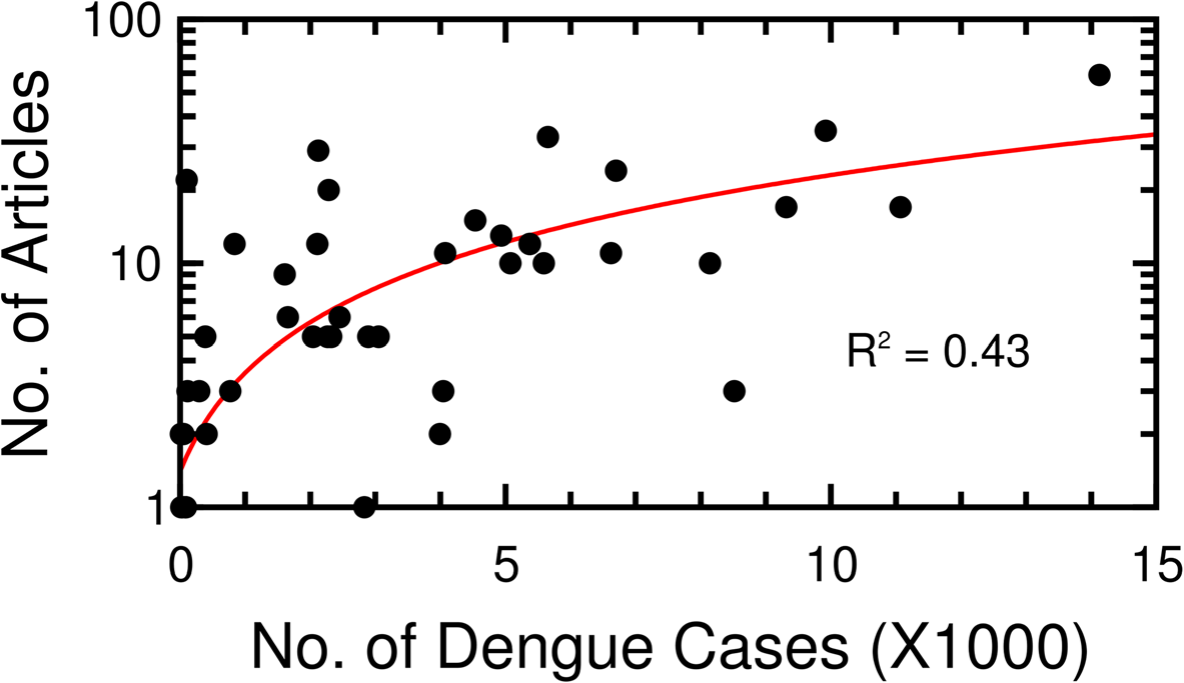
Non-linear correlation in the yearly number of dengue cases in each state in India versus the frequency that each state appeared in the news articles from 2013 to 2016. The red line represents the regression curve with an R^2^ value of 0.43

We used the data mining tool to extract and to record the numbers of dengue-related articles for each state published in the TOI and HT from 2013 to 2016. These data were correlated with the state-wise numbers of dengue cases in Fig. 4 [38].

Overall, visual inspection of the scatterplot together with the relatively low *R*^2^ value of 0.43 indicates a poor correlation between numbers of dengue cases in the states and numbers of articles attributed to those states. In order to identify other factors that may be driving the poor spatial statistics, we conducted a more detailed state-by-state analysis to potentially achieve better correlations. In other words, we hypothesize that state-level correlations would be stronger, suggesting that inherent differences among the states were driving the poor correlations in Fig. 4.

**Fig. 5:**
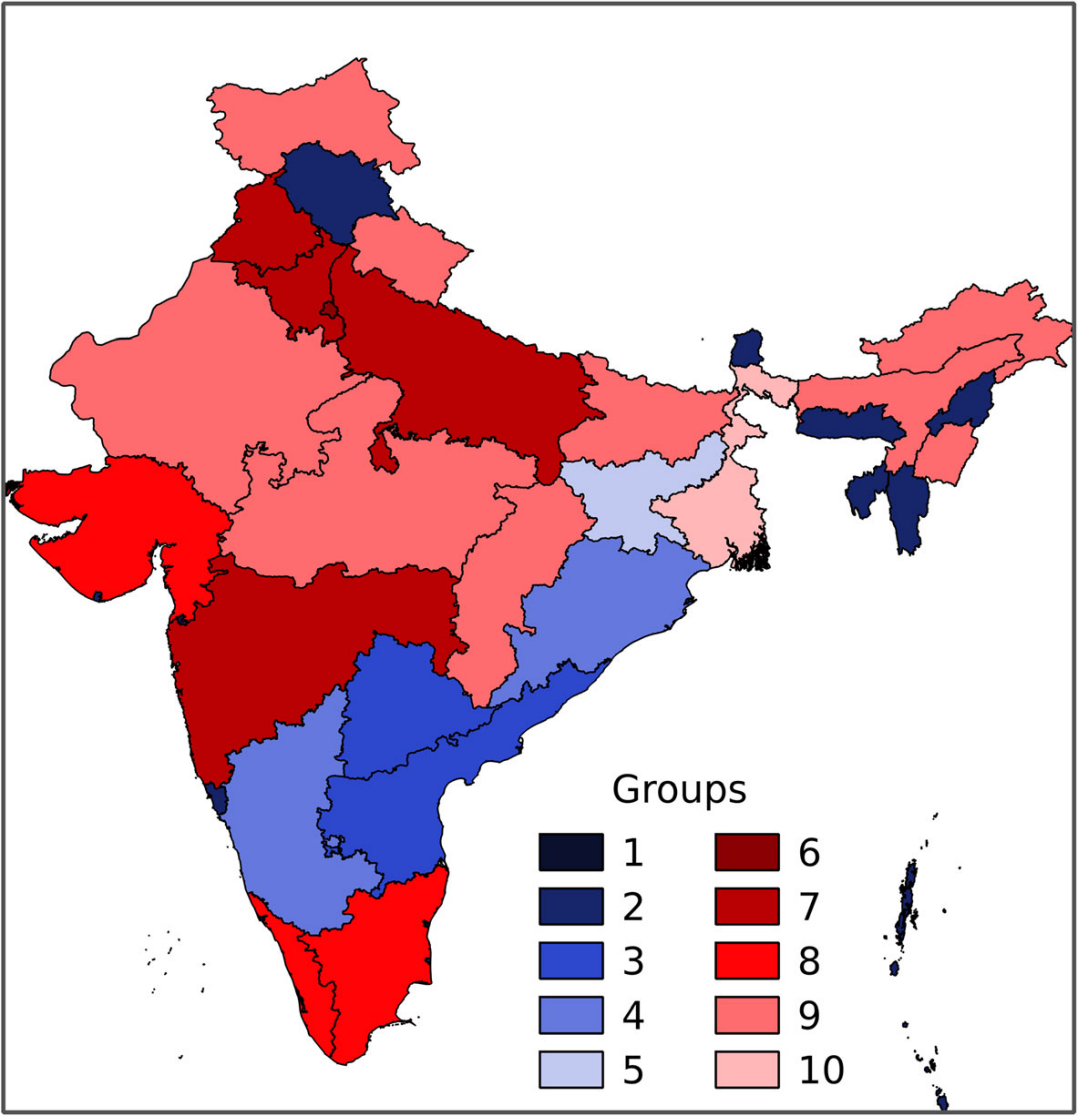
The correlation analyses provided a basis for identifying states and territories exhibiting the strength of correlations between numbers of dengue cases and numbers of news articles for those places. Groups 1 to 5 exhibit poor correlations (blue colors). Groups 6 to 10 exhibit strong correlations (red colors)

For each of the 36 states and union territories, we mined geographic names in each of the articles to determine which state or territory was the focus of the article. Of the 36 states and union territories, nine were never mentioned in any news articles. A further nine exhibited poor correlations between numbers of dengue cases and numbers of articles (*R*^2^ < 0.5). The remaining 18 exhibited good correlations with *R*^2^ values > 0.5, including 13 with *R*^2^ values > 0.7. Based on these correlations, as well as associated geographic, climate, and social differences, we separated the states and union territories into 10 groups (Fig. 5). The grouped states and their features are summarized in Table 1.

**Table 1:** Groups and features of states and union territories

Group	States or union territories	Features	Correlation
1	Andaman and Nicobar, Dadra and Nagar Haveli, Daman and Diu, Lakshadweep, Chandigarh, and Chhattisgarh	Small areas (e.g., size of a city) or islands far away from the mainland	Poor
2	Himachal Pradesh, Nagaland, Sikkim, Tripura, Meghalaya, Mizoram	Remote regions (far north or northeast)	Poor
3	Telangana and Andhra Pradesh	Inconsistent dengue data (states reorganized in 2014)	Poor
4	Karnataka and Orissa	Coastal areas, consistent severe outbreaks	Poor
5	Jharkhand	Consistently minor outbreaks	Poor
6	Delhi	Largest reported dengue cases, greater media interests, large population density, political importance	Good
7	Punjab, Haryana, Uttar Pradesh, and Maharashtra	Close to Delhi, relatively large dengue cases and news reports	Good
8	Tamil Nadu, Kerala, and Gujarat	Coastal areas, high rainfalls, moderate dengue cases, relatively small numbers of news reports	Good
9	Bihar, Jammu and Kashmir, Uttarakhand, Arunachal Pradesh, Assam, Manipur, Rajasthan, Madhya Pradesh, and Uttar Pradesh	North or west arid areas, small numbers of dengue cases and news reports	Good
10	West Bengal	Large dengue cases, but relatively few reports	Good

The spatial analyses of dengue fever outbreaks (e.g., Table 1) provide a starting point to elucidate possible linkages between the disease and multiple factors, such as population density and precipitation. In order to develop these relationships, we analyze five selected states, Delhi, Punjab, Bihar, Gujarat, and Tamil Nadu. Selection for this analysis involved meeting multiple criteria: (1) they are among the 18 states where good correlations exist between dengue cases and number of news articles, so that it is appropriate to represent dengue outbreaks using article numbers; (2) the numbers of articles each month are appropriately high to contribute to a four years temporal analysis (2013–2016); (3) they experience severe dengue outbreaks every year; and (4) the collection of selected states provide variability in terms of location, rainfall amounts, temperature, or population densities.

**Fig. 6:**
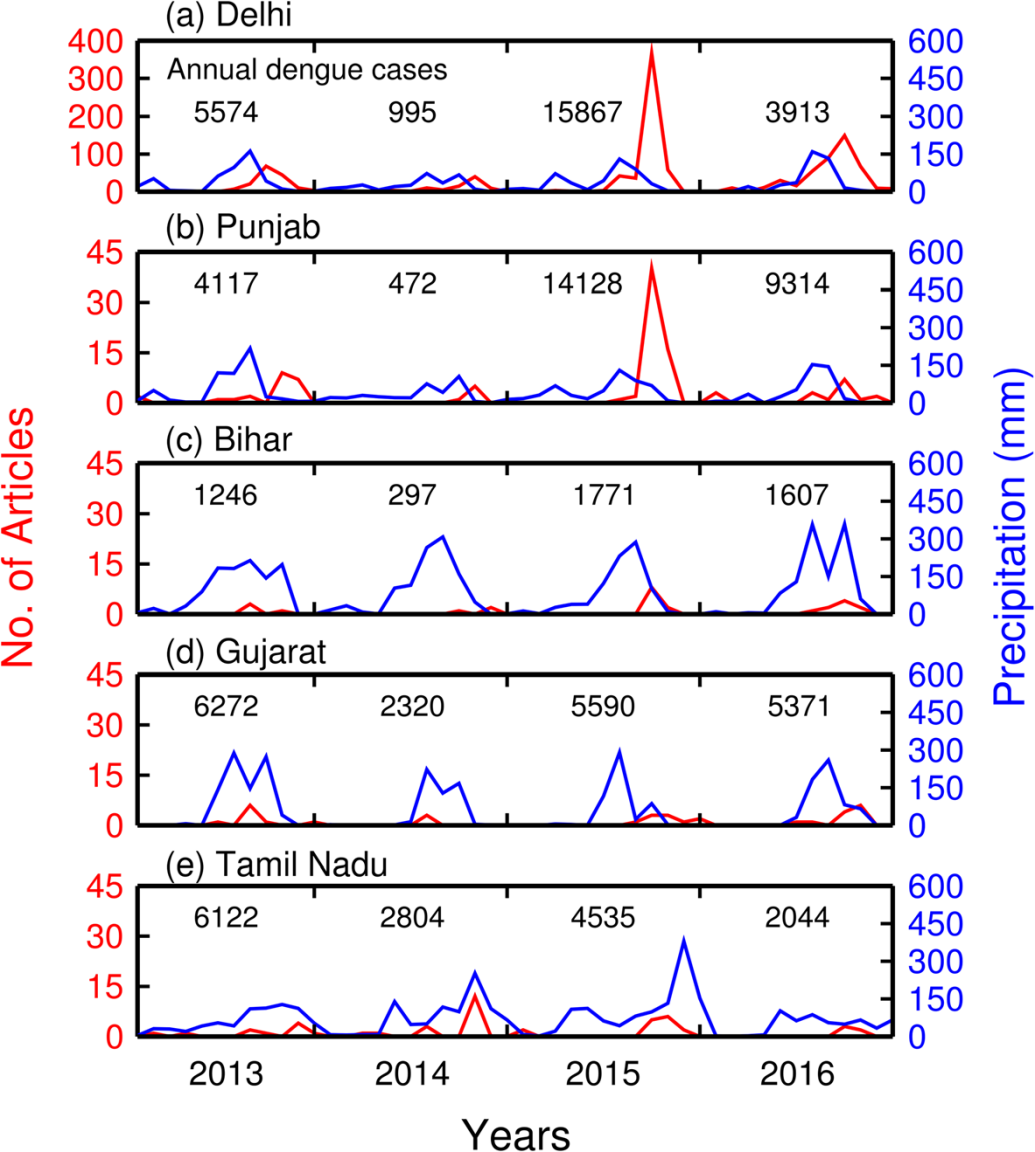
The monthly number of news articles in five states compared with monthly rainfall between 2013 and 2016. For comparison, the actual numbers of disease case for each year are also included

Using four years of data (2013–2016), we compare the monthly number of news articles with precipitation for each of these five states in Fig. 6. All the figures are presented using same scale for both the *x* and *y* axes, except for the number of articles in Delhi. The numbers of news reports for Delhi are much higher than other states and territories.

Figure 6 shows generally that precipitation and the numbers of news reports concerned with dengue fever both fluctuate in concert during the year. However, the states each exhibit marked differences in the quantity and duration in annual precipitation, patterns of precipitation, and numbers of news articles. For example, the drier states, Delhi and Punjab are commonly dry for much of the year except for monsoon rains, occurring between June and August. In these states, the numbers of news reports usually increase following the peak of rainfall with a 2-month time lag.

Bihar and Gujarat are the wettest states with greater cumulative monsoonal rainfalls with a longer duration as compared to Delhi and Punjab. In both Bihar and Gujarat, there is no consistent tendency for dengue to develop following monsoonal rains. The relatively small number of articles there mostly reflected the timing of the onset of infections but not the duration of monsoon periods (Fig. 6c, d).

Tamil Nadu (Fig. 6e) exhibits yet another pattern of dengue association with monsoon rainfall. The rainfall there has less monthly variability than either Bihar or Gujarat, and persists over about 9 months every year (Fig. 6c, d). What is more important, dengue outbreaks have two peaks in Tamil Nadu annually (Fig. 6e). We will examine this special pattern in the discussion section.

### Spatial variances of newsworthiness

Besides factors such as climate and population, we also expected that general practices followed by news media are influential as well. For example, large national newspapers focus predominately on nationally important issues, e.g., politics, geopolitical problems, health, business, and more. They also examine issues of concern for their core, urban readers. We see this pattern with TOI and HT where problems of dengue fever in Delhi are more intensively reported as compared to other places. As far as interest in dengue is concerned for places away from Delhi, we would expect Delhi newspapers to be less interested in problems in those places. A story would need to rise to an increasingly higher threshold before a story would be considered newsworthy for the Delhi readers.

**Fig. 7:**
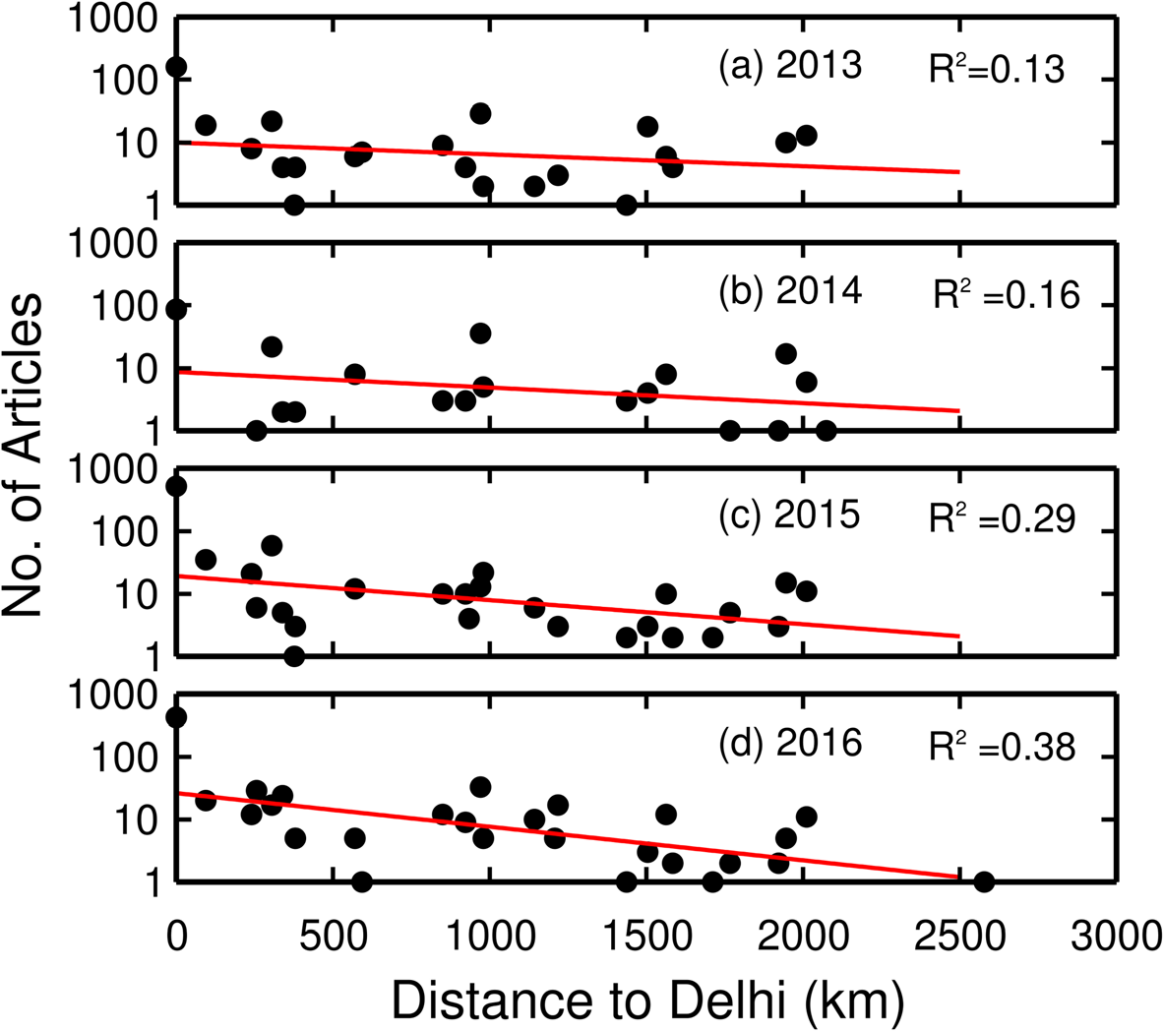
Variations in the numbers of news reports concerned with dengue in each state versus the distances from states to Delhi. The red lines show a consistent tendency for article numbers to decline away from Delhi.

We test the hypothesis that places (states) farther away from Delhi are less well represented in news reports. In this analysis, the distance from the center of each state to Delhi was measured using Google Earth and correlated with the frequency of dengue-related articles for each state across four successive years (2013–2016). Figure 7 shows that the newsworthiness of news articles relating to regions away from Delhi decreases when its distance away from Delhi increases. This result explains why only a few news articles (average 5 reports annually) were published for states located farther away in northeast and northwest India, e.g., West Bengal, even though the number of dengue cases was relatively high (average 7,500 cases annually) (Fig. 5, Groups 2 and 10).

We examined these data in more detail to determine whether differences might exist among states in the four different years. Given the distribution of data points on Fig. 7, we subdivide the collection of points into two families, those states less than 600 km away from Delhi and those more than 600 km away. The reason for subdividing the data in this manner is that up to 600 km away from Delhi, the numbers of reports decreased sharply with distance. However, more than 600 km away from Delhi, the fall-off in numbers of articles with distance was much less evident.

**Fig. 8:**
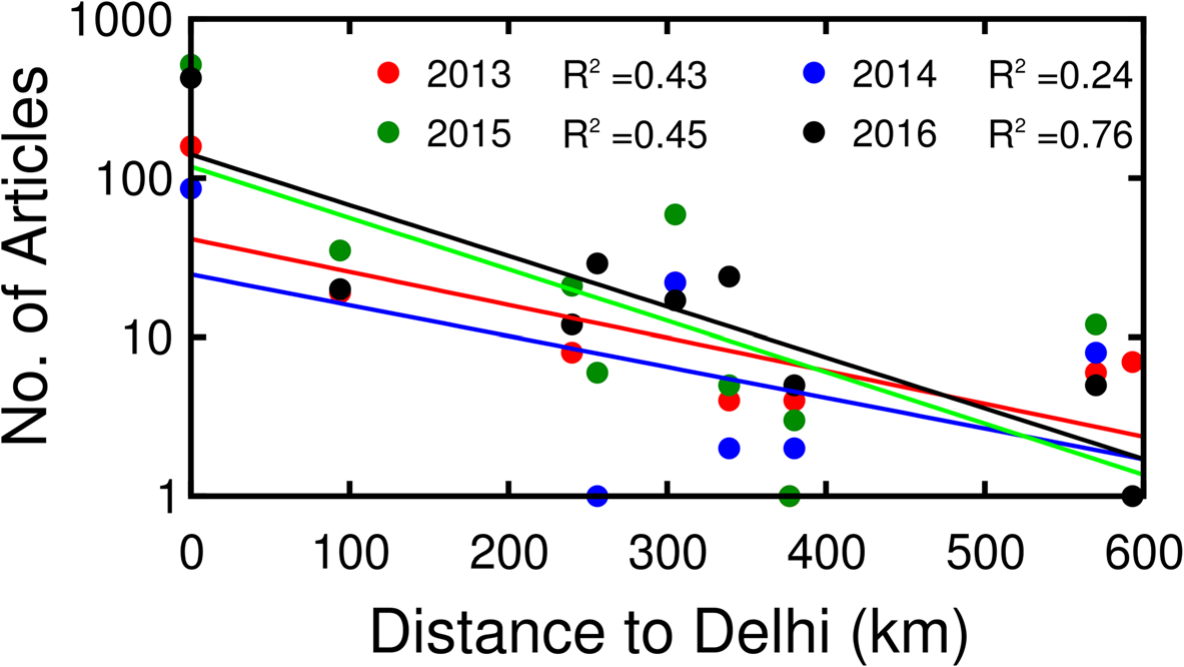
Variations in the numbers of news reports concerned with dengue fever for states within 600 km of Delhi as a function of their distance to Delhi. The dots represent numbers of news reports for the states in 2013–2016. The curves are the trend lines for the 4 different years.

Data for reporting locations closer to Delhi are plotted together in Fig. 8. The straight lines represent the fitted linear regressions in numbers of articles, all of which exhibit a decrease as function of distance for each of the 4 years. The trend lines for 2015 and 2016, years of severe dengue outbreak are steeper than for 2013 and 2014, years notable for fewer dengue cases. Although the logarithmic fits are not strong, they point to a decline in the numbers of articles by about two orders-of-magnitude over 600 km. Inspection of the actual data at Delhi (0 km) and points 575 to 600 km away in Fig. 8 is also instructive. At Delhi, there is an approximately 6× increase in numbers of articles from 86 in 2014 to 515 in 2015. At 575–600 km, the range for the five data points shown there are fewer articles. But more importantly the small number of articles (~ 10) means the statistical power for prediction is poor as is evident by inspection. Data (not shown) shows that further away there are typically 1–10 articles per year without any clear separation in intensity of dengue fever among the years.

## Discussion

Using information related to dengue, our study illustrates how information extracted from news articles provides a useful way to study the epidemiology of this disease. Our previous study [2] showed how the weekly fluctuations in numbers of news articles from two national newspapers can be used to monitor the temporal trends of dengue outbreaks in India. Strong correlations resulted from comparisons of the reported numbers of dengue cases with the numbers of news articles both in multi-year and yearly analyses. However, the study relies on the information extracted from a large collection of various news reports, which are concerned mainly with issues around large dengue outbreaks. For more sporadic and limited dengue cases, our approach is less sensitive.

Because dengue is a mosquito-borne infectious disease, the dynamics of dengue outbreaks are strongly influenced by monsoonal precipitation, which serves to amplify the mosquito populations and hence outbreaks of dengue (Fig. 1) [22]. However, there is often a time lag between the peaks of monsoonal rainfall and the increases in case numbers of dengue. High water flows during the monsoon may cause mosquito eggs to be washed away. There is a much greater opportunity for mosquito numbers to increase after the monsoons with a time lag when abundant stagnant water bodies still exist [39]. In addition, the peak in disease cases is pushed back because the dengue virus experiences extrinsic and intrinsic incubation periods in mosquitoes and human bodies, respectively. The incubation periods are 5–15 days in mosquitoes and 4–10 days in humans [40, 41]. Finally, some time is required for dengue to progress to symptoms that are more serious.

As a result, as mosquito numbers explode in wet conditions associated with the waning monsoon, dengue outbreaks increase as well [42], reaching a peak during September or October with a 1–2-month time lag following the monsoon season. Epidemic dengue fever typically persists for 3 to 5 months with case numbers declining in October or November [43]. This pattern is expressed well for Delhi and the surrounding states, e.g., Punjab. The great increase in stagnant water bodies through the monsoon leads to commensurate increases in the numbers of mosquitoes.

This pattern of association of dengue with wet periods, however, is not the same everywhere. Both the reported dengue cases and numbers of news articles showed a variety of different behaviors that reflect local variability beyond the general pattern just mentioned. The major considerations are rainfall, population, and location. For example, the states with larger population densities usually experience more severe dengue outbreaks (on a case per 1000 basis). Delhi and its populous neighboring states (Fig. 5, Group 6, 7, and 10), are good examples of places where outbreaks are severe. Alternatively, states located in the mid-west, e.g., Rajasthan, Madhya Pradesh, and Uttar Pradesh (Fig. 5, Group 5 and 9), have fewer dengue cases due to their arid climate (Additional file 1: Figure S3).

Annual precipitation in southern coastal regions is longer in duration and larger in quantity (commonly > 150 cm) than the northern interior states, especially during the monsoon season (Additional file 1: Figure S3, also refer to Additional file 1). More rain typically leads to greater numbers of mosquitoes [21]. Among these states in coastal areas, Tamil Nadu is a unique example. Dengue outbreaks there are longer in duration with two peaks annually (Fig. 6e). A study by Tewari et al*.* confirmed the presence of *Aedes aegypti* mosquitoes year round, even through the dry season, approximately January through April. Mosquito numbers are reasonably well correlated to rainfall amounts [44]. A study by Muthu Ramakrishnan and Jenathunnisha found the numbers of medically determined cases of dengue fever plotted monthly for 2012 began increasing in March to a first peak in June, followed by a hiatus in July and August, before rising to a maximum in October and zero by December [45]. This behavior is different from other northern states with the summer or autumn surge in case numbers. Interestingly, the time series in news articles for Tamil Nadu (Figure 6e) captures the tendency for cases to be spread through a year, e.g., 2013 and 2014 and the largest peak in case numbers to be late in year (2014–2016). However, the analysis is not particularly robust because of the relatively small numbers of articles.

The occurrence of dengue fever is also well-known to be not only associated with environmental factors but also patterns of local immunity to the various disease strains [46]. However, the environmental drivers are noticeable with evident correlations. Most importantly, the numbers of dengue cases are lowest during drier periods for both the drier and wetter regions (Fig. 6). There are hints as well in the data that a necessary condition for epidemics to develop are wet years. This issue, however, is a limitation of this study, and will require further work.

Our study also examined the possibilities of using the actual textual information from the news articles to elucidate spatial patterns in cases of dengue fever. Several important results were apparent from this analysis. First, the geographical patterns of news reporting with respect to dengue fever are not homogeneous for the entire country. For example, the TOI and HT do not set out to make sure all parts of India are represented “fairly” in the news that they carry. This spatial variability helps explain why the correlations in Fig. 4 that related news reports for individual states with case numbers tended to be weak.

These observations naturally lead to a second important result, namely that article numbers collected on a state-by-state basis are not the same as a constructed monitoring system that is designed to provide representative data in a way that minimizes various biases. Our study suggests that there are always a large number of news articles reporting on issues of dengue in the city of Delhi, the political center of India, as well as the surrounding states (e.g., Punjab, Haryana, etc.). The states along southwest coast carry a large dengue burden in terms of cases. Yet, there are not many news reports from those areas. Because dengue always is present to some extent in those areas, there is little information for the newspapers to report that is new or newsworthy. During the severe dengue years (e.g., 2015 and 2016), the large problems in and around Delhi were obviously newsworthy with newspapers clearly preoccupied with local conditions there. Being the capital region of India also meant that there would be articles outlining the different ways in which the government was working to deal with the epidemic. In other words, there would be information dealing government assessments, measures adopted by the government, and new medical advancements in treatments, drugs, etc.

This tendency to focus news directly affecting their core Delhi readership, especially during severe outbreaks, appears to occur at the expense of other areas in India. This implicit bias in the dengue data reporting adds complication to spatial applications of textual information. Overcoming this major limitation of “local Delhi bias” in information will require more advanced approaches that can yield interpretations that properly reflect the choices that newspapers make in their reporting.

## Conclusions

The paper describes a follow up our initial study that applies newspaper articles to study outbreaks of infectious diseases [2]. It has examined the possibilities of applying extracted text information from news articles to study the epidemiology of infectious diseases, using dengue fever outbreaks in India as an example.

Both temporal and spatial analyses of dengue fever are conducted by examining information in the actual text of news articles concerned with dengue and India. In terms of temporal aspects, dengue outbreaks generally follow consistent patterns every year. Commonly, dengue infections begin to increase, following monsoon rains from June through August, to reach a peak in September and October with a 1–2-month time lag. In a number of key states mostly in and around Delhi, the numbers of news articles are strongly correlated with patterns of dengue outbreaks in both multi-year and yearly intervals, with both regional and local scales.

Spatially, there are some differences in the way dengue outbreaks are manifest, for example, among different states of India. These differences are determined by complex factors, such as climate, population, and medical conditions. Generally, dengue outbreaks are more severe for (1) the states along the southwestern coast; (2) northeastern states along coast of the Bay of Bengal, which is close to Bangladesh; and (3) Delhi and surrounding states. A closer look at the news articles revealed interesting geographical patterns in news reporting with respect to dengue. Essentially, article numbers are not homogeneous across the entire country. Delhi and its surrounding states always attract more attentions from news reports with respect to dengue. This is probably because of the large numbers of people there and the tendency for dengue problems to be magnified. Moreover, Delhi is ground-zero for most of the government efforts to ameliorate problems across the country.

While there are possibilities for estimating spatial variability in dengue fever using news reports, the results to date have been mixed. News articles have proven useful in examining annual patterns of dengue fever. However, for many areas this application is frustrated by the relatively small numbers of news articles. The main benefit of this study is showing the potential of this approach with possibilities to make better use of information extracted from news articles to overcome the limited numbers of articles and inconsistencies in their choices of what to print. As a conclusion, more advanced approaches are required to produce interpretations that provide for richer interpretations of information provided in the article. In our future research, other non-climatic factors, such as demographic, socio-economic, and other associated risk factors affecting the occurrence of dengue cases, will be examined.

## Supplementary information

**Additional file 1: ** Basic Information of India. **Table S1.** List of Indian States and Union Territories. **Figure S1.** Map of Indian States and Union Territories. **Figure S2.** Map of Indian population density. **Figure S3.** Averaged annual rainfall map of India (2013-2016). The red arrows are monsoon move directions during summer.

## Data Availability

The datasets of extracted news articles during the current study are available in the LexisNexis Database. https://www.lexisnexis.com/communities/academic/w/wiki/30.lexisnexis-academic-general-information.aspx. Indian rainfall data from 2013 to 2016 is obtained from Rainfall Statistics of India Reports published from India Meteorological Department, Ministry of Earth Sciences, http://hydro.imd.gov.in/hydrometweb/(S(c5a3ea55yicst2auwl5ak255))/PRODUCTS/Publications/Rainfall Statistics of India - 2013/Rainfall Statistics of India - 2013.pdf. http://hydro.imd.gov.in/hydrometweb/(S(kslpgz45u3xjiyj04caxljiz))/PRODUCTS/Publications/Rainfall Statistics of India - 2014/Rainfall Statistics of India - 2014.pdf. http://hydro.imd.gov.in/hydrometweb/(S(5ssokh45alcbz345e5jcvg55))/PRODUCTS/Publications/Rainfall Statistics of India - 2015/Rainfall Statistics of India - 2015.pdf. http://hydro.imd.gov.in/hydrometweb/(S(0ymurl55bikbhgzupnyvnny0))/PRODUCTS/Publications/Rainfall Statistics of India - 2016/Rainfall Statistics of India - 2016.pdf. The data of population densities of the states and territories in India is from Census of India 2011. http://censusindia.gov.in/2011-prov-results/paper2/prov_results_paper2_india.html.

## Abbreviations

DENV: Dengue virus
HT: Hindustan Times
TOI: The Times of India
