# Supplementary material for: Disease surveillance using online news: an extended study of dengue fever in India
Source: Trop Med Health. 2019 Dec 11;47:58. doi: 10.1186/s41182-019-0189-y (PMC6905009; doi:10.1186/s41182-019-0189-y)
Supplement: Supplementary file 1 — Additional file 1: Basic Information of India. Table S1. List of Indian States and Union Territories. Figure S1. Map of Indian States and Union Territories. Figure S2. Map of Indian population density. Figure S3. Averaged annual rainfall map of India (2013-2016). The red arrows are monsoon move directions during summer. [file 41182_2019_189_MOESM1_ESM.zip › table S1.docx]

| **No.** | **States** | **No.** | **Union Territories** |
| --- | --- | --- | --- |
| 1 | Andhra Pradesh | 1 | Andaman and Nicobar Islands |
| 2 | Arunachal Pradesh | 2 | Chandigarh |
| 3 | Assam | 3 | Dadra and Nagar Haveli |
| 4 | Bihar | 4 | Daman and Diu |
| 5 | Chhattisgarh | 5 | Lakshadweep |
| 6 | Goa | 6 | Puducherry |
| 7 | Gujarat | 7 | National Capital Territory of Delhi |
| 8 | Haryana |  |  |
| 9 | Himachal Pradesh |  |  |
| 10 | Jammu and Kashmir |  |  |
| 11 | Jharkhand |  |  |
| 12 | Karnataka |  |  |
| 13 | Kerala |  |  |
| 14 | Madhya Pradesh |  |  |
| 15 | Maharashtra |  |  |
| 16 | Manipur |  |  |
| 17 | Meghalaya |  |  |
| 18 | Mizoram |  |  |
| 19 | Nagaland |  |  |
| 20 | Orissa |  |  |
| 21 | Punjab |  |  |
| 22 | Rajasthan |  |  |
| 23 | Sikkim |  |  |
| 24 | Tamil Nadu |  |  |
| 25 | Telangana |  |  |
| 26 | Tripura |  |  |
| 27 | Uttar Pradesh |  |  |
| 28 | Uttaranchal |  |  |
| 29 | West Bengal |  |  |
